# Evaluation of Mobile Health Technology Interventions for the Postdischarge Management of Patients With Head and Neck Cancer: Scoping Review

**DOI:** 10.2196/49051

**Published:** 2023-10-23

**Authors:** Yufei Li, Weihong Chen, Yanjing Liang, Ling Yang, Lili Hou

**Affiliations:** 1 School of Nursing Shanghai Jiao Tong University Shanghai China; 2 School of Nursing Chengdu University of Traditional Chinese Medicine Chengdu China; 3 Department of Nursing Shanghai Ninth People’s Hospital Shanghai Jiao Tong University School of Medicine Shanghai China

**Keywords:** head and neck cancer, mobile health technology, postdischarge, self-management, rehabilitation

## Abstract

**Background:**

Patients with head and neck cancer (HNC) often experience various types and degrees of complications and functional impairment following surgery or radiotherapy. Consequently, these patients require extensive postdischarge rehabilitation, either at home or in the community. Numerous studies have shown the advantages of mobile Health (mHealth) technology in assisting patients with cancer with self-management and rehabilitation during the postdischarge period. However, few reviews have focused on the intervention, management, and evaluation of mHealth technology in postdischarge patients with HNC.

**Objective:**

This study aimed to conduct a scoping review of mHealth technology apps and interventions currently available to patients discharged from hospitals after receiving treatment for HNC. This study sought to identify and summarize the types and effectiveness of existing mHealth interventions as well as the differences in their outcome assessments.

**Methods:**

The PubMed, Embase, Web of Science, and CINAHL databases were used to identify studies with no publication time limits. The keywords “mobile health technology” and “head and neck cancer” were combined to address the main concepts of the research questions.

**Results:**

Of the 1625 papers identified, 13 (0.8%) met the inclusion and exclusion criteria. Most studies (n=8, 61.5%) were randomized controlled trials (RCTs) and cohort studies. These studies were conducted in 6 countries. The main aims of the mHealth interventions in these studies are as follows: (1) symptom monitoring and assessment, (2) rehabilitation training, (3) access to medical health information, (4) telehealth advisers, (5) peer communication and support, and (6) follow-up/review reminders. The outcome evaluations of the 13 included studies were grouped into 4 categories: (1) technology usability and patient satisfaction, (2) self-management of symptoms and patient-reported outcome–related indicators, (3) adherence, and (4) health-related quality of life.

**Conclusions:**

A limited number of studies have investigated the use of mHealth technology in the postdischarge self-management of patients with HNC. The existing literature suggests that mHealth technology can effectively assist patients with HNC in self-management and postdischarge interventions. It plays an important role in addressing patients’ health information needs, reducing both their somatic and psychological burdens, and improving their overall quality of life. Future research should prioritize conducting additional high-quality RCTs to evaluate the usability and analyze the cost-effectiveness of mHealth technology.

## Introduction

### Background

Malignant head and neck tumors, one of the most common malignancies, are classified according to the site of tumor origin into oral cavity, nasopharyngeal, oropharyngeal, and laryngeal cancers. Among these, head and neck squamous cell carcinoma (HNSCC) accounts for >90% of all head and neck tumors. The latest statistics of the International Agency for Research on Cancer (IARC) GLOBOCAN on global cancer incidence and mortality in 2020 revealed 890,000 new cases of HNSCC, with 450,000 resulting deaths annually [1]. The incidence of HNSCC is continuing to rise and is expected to increase by 30% by 2030, reaching an estimated 1.08 million new cases of HNSCC per year [1].

Surgical resection, radiation, chemotherapy, targeted therapy, and a combination of these therapies are the available treatment options for head and neck cancer (HNC). Patients with various tumor stages and locations receive individualized treatment approaches [2]. However, both surgical treatment and radiotherapy can result in various types and intensities of complications, which can negatively affect patients’ somatic function, outward appearance, and psychological well-being. For example, physical dysfunctions, such as swallowing disorders, mouth-opening issues, and shoulder syndrome, as well as psychological difficulties, such as social and workplace reintegration due to an altered outward appearance, can result from both surgical treatment and radiotherapy [3].

Studies have shown that patients with HNC frequently experience increased functional impairment and negative side effects after surgery or radiotherapy. Short-term home rehabilitation after discharge is crucial for enhancing patients’ function and long- and short-term quality of life (QoL) because brief rehabilitation care treatment during hospitalization is insufficient to assist patients in achieving full recovery. Although patients have continuous access to medical care and guidance from doctors and nurses while they are in the hospital, they must assume responsibility for their own functional rehabilitation and self-care after discharge, whether they return home or move to other facilities, such as community nursing homes. Most patients and their family caregivers lack a medical background, and despite receiving necessary verbal health education or health information booklets by doctors and nurses before discharge, forgetfulness inevitably occurs over time. As a result, survivors of HNC may continue to experience significant debilitating issues with swallowing, speech, hearing, and psychological effects due to loss of function and changes in their body image as a result of treatment. Survivors of HNC often experience a lower QoL [4-6]. Studies have shown that their QoL is lower compared to survivors of other cancer types [7]. Therefore, improving the ability of patients with HNC to manage their own care after leaving hospital is a challenging and complex research subject.

With the increasing application and popularity of mobile health (mHealth) technology, numerous studies have shown that mHealth technology has the potential to assist patients with cancer and other chronic diseases in self-management [8,9]. mHealth is defined as “medical and public health practice through the use of mobile devices such as mobile phones, patient monitoring devices, personal digital assistants (PDAs) and other wireless devices” [10]. Currently, mHealth technology is used in a variety of devices and formats, including telephones, mobile phones apps for calls or videos, web-based platforms, and tablets, which are more commonly used in home environments. Although existing studies have systematically evaluated the use of mHealth technology in patients with HNC, the primary focus of these studies is not on patients’ self-management after hospital discharge. The included studies mainly include randomized controlled trials (RCTs), excluding other relevant studies [11]. Therefore, this study aimed to explore how patients can use mHealth technology for self-management and functional rehabilitation, along with assessing the associated outcome indicators, in both the short and the long term, following discharge from treatment.

### Objectives

This study systematically reviewed the use of mHealth technology in the postdischarge self-management of patients with HNC. The review centered on 2 sections: intervention and outcome evaluation. The aims of this study were (1) to summarize the categories of mHealth interventions and their main types of functions/services for the postdischarge self-management of patients with HNC through a systematic review of the existing literature and (2) to examine how these mHealth interventions are evaluated and the differences that exist between outcome indicators across studies.

## Methods

### Study Design

This study used the 5-stage methodological framework outlined by Arksey and O’Malley [12] to define the scope of the review: (1) identifying the research question, (2) identifying relevant studies, (3) selecting relevant papers for the review, (4) charting the data, and (5) collating, summarizing, and reporting the results.

#### Stage 1: Identifying the Research Question

The study population included adult patients with HNC who had been discharged from the hospital after surgical treatment or radiotherapy and were recovering at home or in the community. The type of intervention involved the use of mHealth technology. The research questions were developed based on an initial literature search and further refined through iterative discussions within the research team. The research questions were as follows: (1) What mHealth technologies exist to support patients with HNC after hospital discharge? (2) How are these mHealth technologies used to implement interventions? (3) How are mHealth interventions evaluated?

#### Stage 2: Identifying Relevant Studies

A systematic search strategy was used to identify the literature related to the research questions. We combined the keywords “mobile health technology” and “head and neck cancer” according to the patient/problem, intervention, comparison, and outcome (PICO) principles of literature search, which identified the 2 main concepts of the research questions and summarized the subject terms and free words related to these 2 main concepts. Systematic searches were conducted in the PubMed, Web of Science, Embase, and Cumulative Index to Nursing & Allied Health (CINAHL) databases. Two independent researchers (authors LYF and CWH) searched the databases for references to identify papers published between the time of database creation and March 1, 2023. The search terms and strategy used are presented in Multimedia Appendix 1.

#### Stage 3: Selecting Relevant Papers for the Review

The inclusion and exclusion criteria for this review are presented in Textbox 1. The citations obtained from each database were imported into Endnote Reference Manager for bibliographic analysis. Duplicate papers were excluded. Each level was assessed by 2 reviewers, who independently considered studies based on the inclusion and exclusion criteria. The first screening process involved reviewing the titles and abstracts to make the following decisions: (1) if at least 1 reviewer agreed with the inclusion criteria or found the abstract or title inconclusive, the study was moved to the second level of screening, and (2) if both reviewers agreed to exclude a study, the study was excluded. Two independent reviewers evaluated the full texts at the second level of screening. Any disagreements between the reviewers were resolved through discussion or by a third reviewer.

**Inclusion and exclusion criteria.**

**Inclusion criteria:**

- Adult patients including both males and females (≥18 years old)
- Patients with head and neck cancer (HNC) who have been discharged from the hospital to recover in a nonmedical setting, such as their homes or communities, after undergoing at least 1 surgical or radiotherapy treatment
- Use of mobile health (mHealth) technology to implement interventions
- English papers only
- Contains at least 1 quantitative result
- Selection of a paper by the same research team (same app) on a specific system for inclusion in the analysis

**Exclusion criteria:**

- Participants aged <18 years (pediatric, adolescent)
- Integrated interventions that do not use mHealth technology alone but in other collaborative ways, such as multidisciplinary cooperation
- Full-text documents not available, such as conference abstracts or protocols, as well as review papers
- No specific outcome evaluation indicators related to HNC

#### Stage 4: Charting the Data

We collaborated to develop a graphical form of the data and to identify variables to be extracted. Descriptive graphical information included (1) a general description of the paper (first author and year, study design, study site, and patient population) and (2) intervention-specific information (purpose of the intervention and mHealth app, key features, delivery methods, duration and follow-up period, data collected, outcomes measured, and findings); see Multimedia Appendix 2.

#### Stage 5: Collating, Summarizing, and Reporting the Results

General descriptions of review papers were collated according to the descriptive characteristics. Following a concurrent review of the chart data, we conducted a thematic content analysis of the interventions and associated outcomes for each study. First, codes were developed and applied to analyze the data. The coding segments for all chart data were created using color-coded quotes. The code summaries were organized in a Microsoft Excel table for thematic content analysis. The table was sorted by code and density, looking for repeating patterns addressed by the included papers, including a comparison of the studies across the data set and within each study until key themes were identified. The results of this study summarize the objectives.

## Results

### Search Results

**Figure 1:**
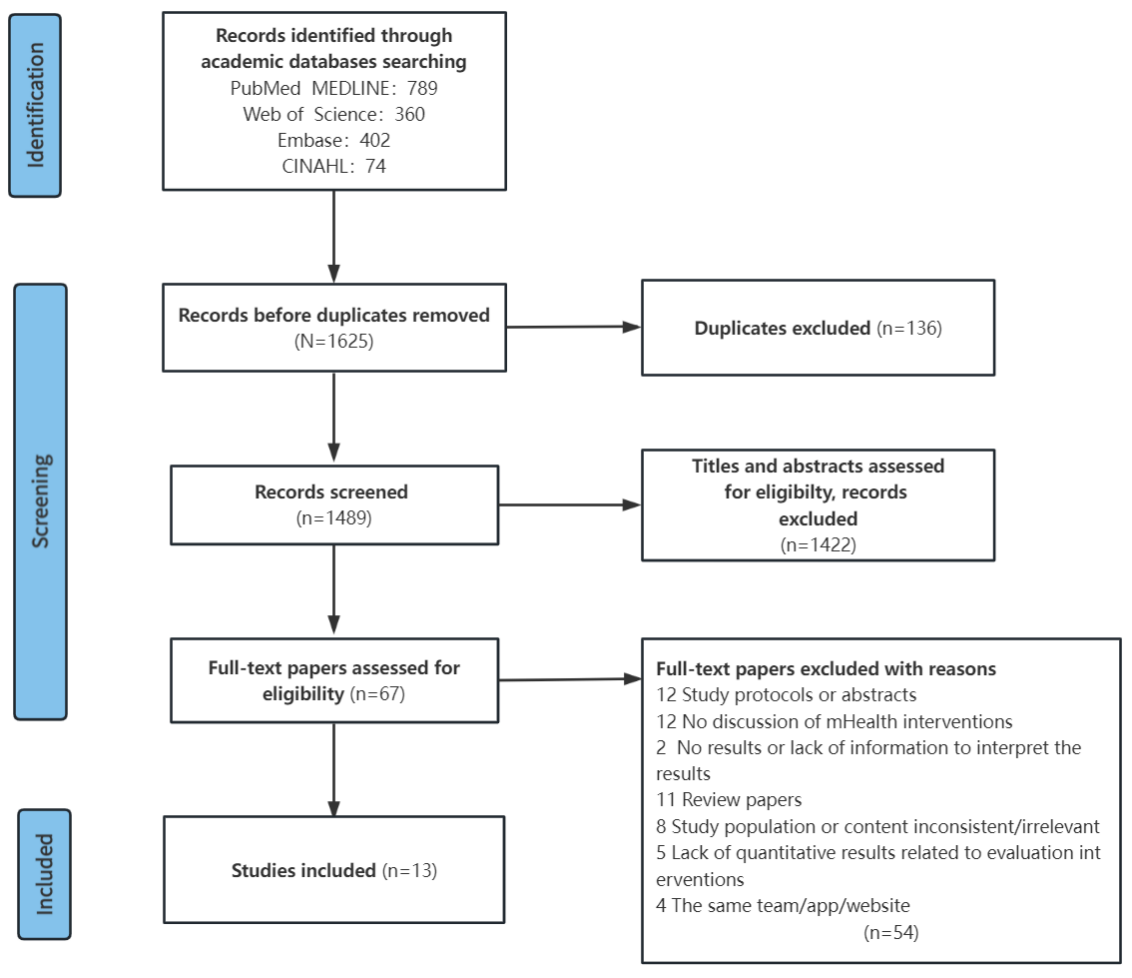
The Preferred Reporting Items for Systematic Reviews and Meta-Analysis (PRISMA) flow diagram.

In total, 1625 papers were retrieved, of which 1489 (91.6%) different papers remained after removing duplicate data. Figure 1 displays the Preferred Reporting Items for Systematic Reviews and Meta-Analyses (PRISMA) flow diagram of the retrieved literature, the level of screening, and the included studies. Finally, 13 (0.9%) studies, projects, or reports were included in this review.

### General Characteristics of the Included Studies

Of the 13 studies included in this review, RCTs (n=6, 46.2%) were the most common type [13-18], followed by 2 (15.4%) cohort studies [19,20] and 5 (38.5%) other types (n=4, 80%, quasi-experimental studies and n=1, 20%, mixed study) [21-25]. The quality of the included studies was assessed using the JBI critical appraisal tools, and detailed information is provided in Multimedia Appendix 3. The general characteristics of the included studies are presented in Table 1.

**Table 1:** General characteristics of the included studies (N=13).

Characteristics		Studies, n (%)
**Study design**		
	RCT^a^	6 (46.2)
	Cohort study	2 (15.4)
	Others	5 (38.5)
**Origin of study**		
	United States	5 (38.5)
	Netherlands	2 (15.4)
	Taiwan	3 (23.1)
	China	1 (7.7)
	Germany	1 (7.7)
	Australia	1 (7.7)
**Year of publication**		
	2007-2019	4 (30.8)
	2020-2023	9 (69.2)

^a^RCT: randomized controlled trial.

### Types of mHealth Interventions Currently Being Implemented

Current mHealth interventions and management services for the population with HNC postdischarge include 6 main categories: (1) symptom monitoring and assessment reports (n=8, 61.5% [15,16,19,20,22-25]), (2) home rehabilitation training (n=4, 30.8% [13,16-18]), (3) medical health information access (n=7, 53.8% [13,14,16,17,22-24]), (4) telehealth support (n=7, 53.8% [14,18-21,23,25]), (5) peer-to-peer communication (n=2, 15.4% [23,24]), and (6) follow-up/review reminders (n=2, 15.4% [14,22]). These mHealth interventions were delivered through various technology platforms, including smartphones (n=9, 69.2%), personal digital assistants (PDAs)/tablets (n=2, 15.4%), web-based platform (n=1, 7.7%), and home monitoring or telemonitoring unit or telemetry system (n=1, 7.7%). The results are summarized in Table 2.

**Table 2:** mHealth^a^ intervention tools and categories.

Characteristics		Studies (N=13), n (%)
**mHealth intervention tools**		
	Smartphone	9 (69.2)
	Tablet or computer	2 (15.4)
	Web-based portal	1 (7.7)
	Home monitoring or telemonitoring unit	1 (7.7)
**mHealth intervention categories**		
	Self-monitoring and report	8 (61.5)
	Home practice	4 (30.8)
	Provision of health information	7 (53.8)
	Telemedical support	7 (53.8)
	Communication community	2 (15.4)
	Follow-up/review reminders	2 (15.4)

^a^mHealth: mobile Health.

Intervention evaluation indicators for patients with HNC adopting mHealth technology mostly included general patient characteristics, QoL indicators, adherence, symptom self-reporting, patient satisfaction, and evaluation of technology usability. The thematic content analysis identified 4 main themes for outcome evaluation: (1) technology usability and patient satisfaction, (2) indicators related to self-management of symptoms and patient-reported outcomes (PROs), (3) adherence, and (4) health-related quality of life (HRQoL).

This study found that mHealth technology plays a more beneficial role in the postdischarge management of patients with HNC. The mHealth program helped improve physical and psychological symptoms after discharge, enabled patients to gain more knowledge about health care, and enhanced their self-management of behaviors and functional rehabilitation exercises after leaving the hospital. Patients in these studies showed high levels of satisfaction with and acceptance of mHealth technologies.

#### Self-Management of Symptoms and PRO-Related Indicators

Of the 13 included studies, 8 (61.5%) focused on the impact of mHealth technology on symptoms and the validity of outcome reporting.

Of these 8 (61.5%) studies, 2 (25%) discussed the impact of mHealth technology on the effectiveness of PROs. Ma et al [20] found that using a fully automated interactive chatbot to provide support and assist patients in self-reporting outcomes during the postradiotherapy period for HNC treatment resulted in good consistency between PROs and clinician-reported outcomes (CROs). According to the study results [20], 61% of the patients felt that the chatbot helped with symptom self-management and reduced the need to call the care team, demonstrating that mHealth technology can be effective in helping patients with self-reporting outcomes.

Due to the significant symptom burden associated with HNC, mHealth technology may be beneficial in assisting patients with symptom management. Van Cleave et al [19] developed a web-based platform, Electronic Patient Visit Assessment (ePVA), to explore patients’ reports of symptoms and functional limitations. ePVA can inquire about and document 21 common symptoms and functional limitations associated with HNC and generate an ePVA report. The HNC care team members can use the ePVA report to implement real-time clinical interventions based on their clinical judgment and the patients’ knowledge. The study phase results showed that patients with the most symptoms and functional limitations reported a significantly poorer HRQoL, demonstrating that ePVA may be a proven mHealth tool that can be used as a real-time intervention for patient reporting.

Next, 6 (75%) studies discussed the effect of mHealth technology on patients’ symptom self-management. The mobile app SwallowIT was tailored to provide telepractice-assisted therapy to patients with swallowing disorder during radiotherapy and after HNC treatment to support home practice of the pharyngeal program [13]. The program provides instructional videos, images, and text descriptions for each exercise, allowing patients to record the number of motor repetitions and the perceived effort as they complete each exercise. Results of the study during the outcome evaluation phase showed no significant differences between the conventional and experimental groups in terms of swallowing, nutrition, or functional measures. However, more patients (76%) preferred using SwallowIT to receive instructions compared to instructing themselves during exercises.

Lin et al [24] developed an oral care mobile app to provide medical health information and oral mucosal care advice to patients during radiotherapy for HNC. The results of the study showed that the Patient-Generated Subjective Global Assessment scores of the group using mHealth technology were significantly lower than those of the control group at all 3 nodes during the intervention. Additionally, the severity of oral mucositis grading was also significantly lower in all groups. These findings indicate that the use of mobile apps is effective in improving the nutritional status and reducing the side effects in patients with HNC treated with concurrent radiotherapy.

The mobile app Oncokompas, developed by Dutch academics, can support symptom self-management by providing medical information and a personalized overview of supportive care options aimed at reducing the symptom burden among survivors of cancer [16]. The results of the study indicated that the mHealth technology group showed statistically significant improvements in the clinical course and symptom burden scores for oral pain, social eating, swallowing, cough, and dental symptoms compared to the control group.

Wang et al [18] used telephone calls to help patients with oral cancer after radical surgery perform functional exercises for their trismus, monitor training progress, and obtain appropriate feedback. After the 12-week intervention period, the change in the maximum interincisional opening was 10.30 mm (95% CI 8.22-12.37) greater in the experimental group than in the active control group. The change in the mandibular functional impairment score was –0.36 (95% CI –0.44 to –0.28) greater in the experimental group than in the active control group. This study provides evidence supporting the effectiveness of the intervention program in reducing dental and mandibular functional impairments in patients undergoing radical oral cancer surgery.

Regarding complication management, an RCT [14] with patients with nasopharyngeal cancer who were discharged from the hospital after radiotherapy used a mobile app to provide the patients with information about the disease, including observation and treatment of radiotherapy complications and reminders for regular review. The aim was to enhance the self-management skills of the patients, enabling them to cope effectively with radiotherapy complications [14]. The results of this study showed that the incidence of oral mucositis, dry mouth syndrome, difficulty in opening the mouth, and nasal congestion was significantly lower in the intervention group than in the control group 5 months after discharge, suggesting that the intervention can effectively help patients improve their ability to cope with and manage complications and reduce their occurrence. In contrast, another retrospective controlled trial [25] was conducted in which patients were followed up over the phone within 72 h of discharge as well as by wound visits to answer their questions in order to reduce emergency department visits and readmission rates. The results of this study showed a statistically significant reduction in emergency department visits compared to the previous year, with no change in readmission rates, demonstrating the potential of telephone interventions in the early postoperative period to reduce unnecessary emergency department visits.

#### Technology Availability and Patient Satisfaction

Of the 13 included studies, 4 (30.8%) investigated technology usability and patient satisfaction in outcome evaluations. Patients demonstrated higher satisfaction with the mHealth technologies in all study outcomes.

In terms of technology usability, software usability refers to the extent to which a particular user can use a product in a specific context and achieve a particular goal effectively, efficiently, and satisfactorily. Patient satisfaction with a fully automated interactive chatbot [20] reached 89%, with 83% of patients finding it easy to use, 79% feeling confident in using the chatbot, 71% finding the chatbot functionality well integrated, and 86% feeling they did not need additional training or technical support (80%) to use the chatbot.

In the SwallowIT study [13], 76% of patients preferred SwallowIT or clinician guidance. In a study [21] on coping with somatic imagery, 89% of patients were very satisfied with BRIGHT, found its use effective, and would recommend it to other survivors of HNC. In a quasi-experimental study in Taiwan [22], the acceptability score of the mHealth app significantly improved (*P*<.05) in terms of the intention to use, perceived usefulness, and ease of use after 3 months of the intervention.

These data show that patients’ use of and familiarity with mHealth apps reduce their uncertainty and improve their acceptance of the new technology. This, in turn, can promote better adoption and use of mHealth technology in the future and enhance its overall usability.

#### Health-Related Quality of Life

Most studies (n=9, 69.2%) measured and evaluated the HRQoL. Of these, 6 (66.7%) studies [14,19,21-24] used the European Organization for Research and Treatment of Cancer Quality of Life Questionnaire Core 30 (EORTC-QLQ-C30) and the European Organization for Research and Treatment of Cancer Quality of Life Questionnaire Core 35 and Head and Neck Module (EORTC-QLQ-H&N35), 2 (22.2%) studies [13,15] used the Functional Assessment of Cancer Therapy—Head and Neck (FACT-H&N) scale, 2 (22.2%) studies [16,19] used the HRQoL scale, and 1 (11.1%) study [17] used the European Organization for Research and Treatment of Cancer Quality of Life Questionnaire and Provisional 25-item Information Module (EORTC-QLQ-INFO-25) to assess the QoL of survivors of HNC.

There were mixed results regarding the improvement in the QoL with mHealth technology in the studies. The mobile app SwallowIT was not associated with statistically significant improvements in the final overall QoL [13]. However, BRIGHT was associated with improvements in the social eating disorder and social contact disorder substructural domains of the EORTC-QLQ-H&N35 [21]. Additionally, a greater number of reported symptoms and functional limitations were associated with lower EORTC-QLQ-C30 overall QoL health scores [19]. Furthermore, 5 (55.6%) studies demonstrated higher overall improvements in the QoL in the experimental group using mHealth technology than in the control group [14,15,22-24], and 1 (11.1%) study reported that increased adherence was associated with a better patient-reported QoL [17].

#### Intervention Adherence

Of the 13 included studies, 5 (38.5%) reported on adherence to the use of mHealth technology. In the SwallowIT app [13], it was found that adherence decreased significantly over time for the entire cohort, with low adherence at 6 weeks in all groups (27%). However, adherence was relatively high in the group with clinician guidance and the group using SwallowIT [13].

The results of an RCT [14] showed that adherence to mouth-opening exercises and nasal rinsing was higher in the intervention group than in the control group at 3 and 6 months postdischarge (*P*<.05). However, changes in adherence within groups were not elaborated. The researchers concluded that mHealth technology can provide pictures and videos to be viewed repeatedly, and all these activities can be performed with the help of relevant videos as a means to improve patient adherence.

The ePVA study [19] showed that 59 of 64 (92.2%) patients completed the ePVA, with 1 or more follow-up visits within the 6-month study window, with high completion rates and adherence. The investigators concluded that the high completion rate results were related to the study team accommodating patients’ needs regarding completion times during treatment, such as allowing patients to delay completion until a convenient time.

The HNC Virtual Coach study [17] conducted as a pre-radiotherapy prophylactic swallowing rehabilitation demonstrated that 80% of patients used the app and over 50% completed at least 1 swallowing exercise per day, with better, although not statistically significant, adherence in the experimental group. Although adherence declined in both groups during radiotherapy, the results showed that higher adherence was associated with a better patient-reported QoL.

Additional support for the intervention exercise program for patients with restricted mouth opening was provided by remote telephone support [18], which showed that at week 12, the experimental group had 299.67 minutes (95% CI 223.44-357.89) more intervention exercise time than the control group. From baseline to week 12, the change in the maximum interincisional opening was 10.30 mm (95% CI 8.22-12.37) greater in the experimental group than in the active control group. This demonstrates that the use of mHealth technology can help enhance patient compliance with intervention programs and, in turn, improve functional impairment outcomes.

## Discussion

### Principal Findings

In this review, 13 papers were analyzed. Some studies have indicated that patients with HNC frequently experience increased functional impairment after surgery. Brief rehabilitation during hospitalization may not be adequate to help patients recover, while long-term home-based rehabilitation after discharge plays a crucial role in improving patients’ function and long- and short-term QoL. Therefore, this study focused on assessing how patients use mHealth technology for self-management, functional rehabilitation, and related outcome evaluation indicators after hospital discharge.

This scoping review summarized the existing literature on mHealth technology for the postdischarge self-management of patients with HNC and the intervention tools, intervention methods, and types of outcome evaluations of mHealth technology in the currently available literature, following a generalization and thematic summary.

### Advantages of mHealth Technology in Postdischarge Self-Management of Patients With HNC

mHealth technology can accelerate patient communication, facilitate home monitoring and self-management, and improve overall patient health. The use of mHealth in the postdischarge self-management of patients with HNC addresses many issues and offers several advantages, including the following:

- Real-time monitoring and tracking: Although patients are the best recorders of their daily health experiences, clinicians and others are unlikely to have full access to patients in their living environments after hospital discharge. Consequently, capturing timely information about patients’ actual experiences, health status, and outcomes can be challenging. mHealth apps can help patients monitor and track their health in real time. Using sensors and devices, patients can measure physiological metrics (eg, heart rate, respiration, blood pressure, and weight) and record them. These data can be shared with doctors to better understand their condition and make necessary adjustments to the treatment plans. Meanwhile, because PROs have become the focus of health care research, scholars have begun to explore whether mHealth technology can be an effective tool for PROs. Findings have shown that the use of mHealth technology can help patients with daily symptom recording and outcome reporting. Furthermore, PROs have demonstrated strong concordance with CROs [20].
- Personalized health management: mHealth technology can provide a platform for patients and their families to obtain different health services in the face of different needs, as well as better intervention and management of patients after they leave the hospital. mHealth apps can provide personalized health management solutions tailored to individual patient differences and needs. The apps can provide customized dietary advice, exercise plans, medication reminders, follow-up reminders, and other functions according to the patient’s condition and treatment plan, thereby helping them effectively manage their health.
- Education and information resources: While patients are receiving treatment within a hospital, doctors and nurses can provide a steady stream of health care support, including daily treatment, health information, and guidance. In contrast, once patients are discharged, although doctors and nurses may initially explain the key points of posthospitalization rehabilitation and care, the passage of time and various factors, both subjective and objective, such as patient compliance, can make it challenging for patients to effectively self-manage their physical and psychological well-being. mHealth apps can provide patients with health education and the necessary information resources they require anytime, anywhere. Patients can access professional medical knowledge about diseases, treatment options, medication, and more through these apps while they are at home, helping them better understand their condition, treatment options, recovery methods, etc.
- Social and psychological support: Due to factors related to the disease and treatment modalities, patients with HNC usually face issues concerning an altered body image, which may also affect their ability to resume work and engage in social activities. Therefore, social and psychological support for these patients requires careful consideration. However, this is a long-term process that cannot be fully addressed and promptly implemented during hospitalization. mHealth apps connect patients with other patients or health care professionals to provide social and psychological support. Patients can use these apps to share their experiences and offer advice to other patients. Moreover, they can have online consultations and communications with health care professionals, thus reducing anxiety and the feeling of isolation.
- Improving adherence: Undoubtedly, for doctors and nurses, managing and providing follow-up care for discharged patients pose a significant challenge. The brief, 1-time health education at the time of discharge makes it difficult to address the various medical, health, and psychosocial difficulties encountered by patients during home rehabilitation and to help patients establish good self-management ability and compliance with home treatment. Patients who leave the hospital often experience problems during the follow-up phase, including difficulty in communication, difficulty in management, poor adherence, suboptimal recovery, and a poor QoL. mHealth apps, however, can provide medication reminders, treatment plan tracking, and other functions to help patients better follow doctors’ advice and treatment plans.

These findings suggest that novel mHealth technology is more likely to be welcomed by the patient population than traditional forms of follow-up and that patients who use mHealth technology show better adherence [13,14,17-19]. This suggests that mHealth technology may be an effective tool to provide follow-up and enhance the self-management of postdischarge patients in the future.

Overall, the advent of mobile technology has transformed the health ecosystem by changing the way individuals communicate and providing patients and health care providers with a wide range of supportive tools to monitor and manage health information, thereby facilitating better health care delivery. The advantages of mHealth in the home self-management of patients with HNC after discharge from the hospital include real-time monitoring, personalized health management, access to educational and information resources, social and psychological support, and improved treatment adherence. These benefits can help patients more effectively manage their health, improve their QoL, and work closely with their health care teams.

### Limitations of mHealth Technology in Postdischarge Self-Management of Patients With HNC

Although most studies have demonstrated the positive impact of mHealth technology on the postdischarge self-management of patients with HNC, some have shown no significant improvement in patient self-management with respect to other outcome indicators [16]. This may be because mHealth technology is just beginning to be applied to the population with HNC and the amount of available literature on its application is limited. Although the number of RCTs among the available studies is relatively high, the research questions and outcome indicators vary significantly across these studies. Therefore, there is a scarcity of directly comparable studies in terms of outcome evaluation.

In addition to physical and psychological outcome indicators, few studies have examined standard technical usability evaluations and economic efficiency indicators. Wall et al [26] reported associated health service costs (service time, consumables, treatment resources), patient-related costs (travel and wages), and patient-related HRQoL statistics. Their study resulted in a comparison of the total costs of different forms of mHealth interventions, revealing significant cost savings for health care services and consumers with HNC, while achieving comparable HRQoL outcomes at a lower cost [26]. However, apart from this study, there is limited existing literature that reports on the social and economic benefits of using mHealth technology for both patients and hospitals. Various factors, such as software development and operational costs, equipment use and maintenance costs, actual changes in patient financial stress, and the overall social and economic benefits to hospitals, are areas where further data and research are needed. Such research is essential to demonstrate the technological advantages of mHealth and the associated individual societal benefits generated.

As the number and scale of mobile apps increase with the increasing use of mHealth technology, more attention should be paid to usability evaluation throughout the software development process. Timely usability evaluation before, during, and after development is an important measure for understanding user needs, improving the design of software features, and improving user experience and satisfaction. However, this is not well described in existing research.

At the same time, we must also consider the impact of mHealth technology on doctors and nurses, even as it brings convenience to patients. Questions regarding whether the use of mHealth technology consumes additional time for health care professionals, whether it increases their workload beyond their regular work, whether health care professionals are satisfied with their mHealth technology experience, and how hospital managers reconcile conflicts and contradictions arising from this technology are all aspects that have received less attention in existing research. Therefore, future research should explore these areas to achieve a comprehensive understanding.

Finally, the issues of data privacy and security should be given sufficient attention. mHealth apps handle large amounts of personal health data; therefore, data privacy and security are important concerns. Ensuring the security and privacy of patient data is challenging, and appropriate security measures must be adopted to protect such data.

### Implications for Future Research

By addressing the limitations and research weaknesses of this study, future research can advance to explore the effectiveness of mHealth technology in the postdischarge self-management of patients with HNC. This can be achieved through rigorous methods, such as conducting an RCT with a robust study design, expanding the scope of the study and the number of users, and adopting scientific theoretical guidance for the selection and evaluation of outcome indicators.

Hotspots for future research will also focus on the application of artificial intelligence in mHealth, the integration of virtual reality and augmented reality technologies in oncology treatment, and the development of remote monitoring and diagnosis based on mobile devices. Future research will also need to overcome various challenges, including issues related to data privacy and security, technology usability and ease of use, data analysis and use, and evaluation of the effectiveness and efficacy of mHealth apps. Addressing these challenges requires interdisciplinary collaboration and sustained research efforts.

### Limitations

This study was a scoping review conducted to broadly include and summarize the existing literature. In addition, this study only included studies published in English, and studies published in other languages were excluded from the discussion.

### Conclusion

mHealth technology has been applied to the postdischarge self-management of patients with HNC. The main interventions in mHealth technology for improving postdischarge self-management include symptom monitoring and reporting, functional rehabilitation training, access to health care information, telehealth service support, peer-to-peer communication, and follow-up/review reminders. Outcome evaluations of the use of mHealth technology were discussed in terms of technology usability and patient satisfaction, indicators related to self-management of symptoms, PROs, adherence, the HRQoL, and the impact on somatic/psychological aspects, with most studies showing a positive impact.

Therefore, based on the limited research data available to date, mHealth technology can effectively help patients with HNC in self-management and postdischarge interventions. This plays an important role in meeting patients’ health information needs, reducing their somatic and psychological burdens, and enhancing their QoL. Future research should aim to conduct higher-quality RCTs for usability evaluation and cost–economic benefit analysis.

## Appendix

Multimedia Appendix 1 Search strategy and search statement.

Multimedia Appendix 2 Overview of studies.

Multimedia Appendix 3 Quality assessment of included studies.

Multimedia Appendix 4 Preferred Reporting Items for Systematic reviews and Meta-Analyses extension for Scoping Reviews (PRISMA-ScR) Checklist.

## Abbreviations

CRO: clinician-reported outcome
EORTC-QLQ-C30: European Organization for Research and Treatment of Cancer Quality of Life Questionnaire Core 30
EORTC-QLQ-H&N35: European Organization for Research and Treatment of Cancer Quality of Life Questionnaire Core 35 and Head and Neck Module
ePVA: Electronic Patient Visit Assessment
HNC: head and neck cancer
HNSCC: head and neck squamous cell carcinoma
HRQoL: health-related quality of life
mHealth: mobile health
PRO: patient-reported outcome
QoL: quality of life
RCT: randomized controlled trial

## References

[1] Sung H, Ferlay J, Siegel RL, Laversanne M, Soerjomataram I, Jemal A, Bray F (2021). Global Cancer Statistics 2020: GLOBOCAN estimates of incidence and mortality worldwide for 36 cancers in 185 countries. CA Cancer J Clin.

[2] Marur S, Forastiere AA (2016). Head and neck squamous cell carcinoma: update on epidemiology, diagnosis, and treatment. Mayo Clin Proc.

[3] Parke SC, Langelier DM, Cheng JT, Kline-Quiroz C, Stubblefield MD (2022). State of rehabilitation research in the head and neck cancer population. functional impact vs. impairment-focused outcomes. Curr Oncol Rep.

[4] Jacot W, Colinet B, Bertrand D, Lacombe S, Bozonnat M, Daurès J-P, Pujol J, OncoLR Health Network (2008). Quality of life and comorbidity score as prognostic determinants in non-small-cell lung cancer patients. Ann Oncol.

[5] Blazeby J, Brookes S, Alderson D (2000). Prognostic value of quality of life scores in patients with oesophageal cancer. Br J Surg.

[6] Thompson TL, Pagedar NA, Karnell LH, Funk GF (2011). Factors associated with mortality in 2-year survivors of head and neck cancer. Arch Otolaryngol Head Neck Surg.

[7] Ganz PA, Lee JJ, Siau J (1991). Quality of life assessment. An independent prognostic variable for survival in lung cancer. Cancer.

[8] Ma Y, Zhao C, Zhao Y, Lu J, Jiang H, Cao Y, Xu Y (2022). Telemedicine application in patients with chronic disease: a systematic review and meta-analysis. BMC Med Inform Decis Mak.

[9] Cox A, Lucas G, Marcu A, Piano M, Grosvenor W, Mold F, Maguire R, Ream E (2017). Cancer survivors' experience with telehealth: a systematic review and thematic synthesis. J Med Internet Res.

[10] World Health Organization (2016). Global Diffusion of eHealth: Making Universal Health Coverage Achievable. Report of the Third Global Survey on eHealth.

[11] Caputo MP, Rodriguez CS, Padhya TA, Mifsud MJ (2023). Telehealth interventions in head and neck cancer patients: a systematic review. Cancer Nurs.

[12] Arksey H, O'Malley L (2005). Scoping studies: towards a methodological framework. Int J Soc Res Methodol.

[13] Wall LR, Ward EC, Cartmill B, Hill AJ, Isenring E, Byrnes J, Porceddu SV (2020). Prophylactic swallowing therapy for patients with head and neck cancer: a three-arm randomized parallel-group trial. Head Neck.

[14] Di R, Li G (2018). Use of a smartphone medical app improves complications and quality of life in patients with nasopharyngeal carcinoma who underwent radiotherapy and chemotherapy. Med Sci Monit.

[15] Pfeifer MP, Keeney C, Bumpous J, Schapmire TJ, Studts JL, Myers J, Head B (2015). Impact of a telehealth intervention on quality of life and symptom distress in patients with head and neck cancer. J Community Support Oncol.

[16] van der Hout A, van Uden-Kraan CF, Holtmaat K, Jansen F, Lissenberg-Witte BI, Nieuwenhuijzen GAP, Hardillo JA, Baatenburg de Jong RJ, Tiren-Verbeet NL, Sommeijer DW, de Heer K, Schaar CG, Sedee RE, Bosscha K, van den Brekel MWM, Petersen JF, Westerman M, Honings J, Takes RP, Houtenbos I, van den Broek WT, de Bree R, Jansen P, Eerenstein SEJ, Leemans CR, Zijlstra JM, Cuijpers P, van de Poll-Franse LV, Verdonck-de Leeuw IM (2020). Role of eHealth application Oncokompas in supporting self-management of symptoms and health-related quality of life in cancer survivors: a randomised, controlled trial. Lancet Oncol.

[17] Starmer HM, Klein D, Montgomery A, Goldsmith T, McCarroll L, Richmon J, Christopher Holsinger F, Beadle B, Jain P (2023). Head and neck virtual coach: a randomized control trial of mobile health as an adjunct to swallowing therapy during head and neck radiation. Dysphagia.

[18] Wang T, Su J, Leung K, Liang S, Wu S, Wang H (2019). Effects of a mouth-opening intervention with remote support on adherence, the maximum interincisal opening, and mandibular function of postoperative oral cancer patients: a randomized clinical trial. Eur J Oncol Nurs.

[19] Van Cleave JH, Fu MR, Bennett AV, Concert C, Riccobene A, Tran A, Most A, Kamberi M, Mojica J, Savitski J, Kusche E, Persky MS, Li Z, Jacobson AS, Hu KS, Persky MJ, Liang E, Corby PM, Egleston BL (2021). The usefulness of the Electronic Patient Visit Assessment (ePVA) as a clinical support tool for real-time interventions in head and neck cancer. Mhealth.

[20] Ma D, Orner D, Ghaly MM, Parashar B, Ames JW, Chen WC, Potters L, Teckie S (2021). Automated health chats for symptom management of head and neck cancer patients undergoing radiation therapy. Oral Oncol.

[21] Graboyes EM, Maurer S, Park Y, Marsh CH, McElligott JT, Day TA, Hornig JD, Sterba KR (2020). Evaluation of a novel telemedicine-based intervention to manage body image disturbance in head and neck cancer survivors. Psychooncology.

[22] Wang T, Huang R, Yang S, Chou C, Chen L (2020). Evaluating the effects of a mobile health app on reducing patient care needs and improving quality of life after oral cancer surgery: quasiexperimental study. JMIR Mhealth Uhealth.

[23] van den Brink JL, Moorman PW, de Boer MF, Hop WCJ, Pruyn JFA, Verwoerd CDA, van Bemmel JH (2007). Impact on quality of life of a telemedicine system supporting head and neck cancer patients: a controlled trial during the postoperative period at home. J Am Med Inform Assoc.

[24] Lin T, Wang Y, Huang C (2022). Effects of a mobile oral care app on oral mucositis, pain, nutritional status, and quality of life in patients with head and neck cancer: a quasi-experimental study. Int J Nurs Pract.

[25] Shah M, Douglas J, Carey R, Daftari M, Smink T, Paisley A, Cannady S, Newman J, Rajasekaran K (2021). Reducing ER visits and readmissions after head and neck surgery through a phone-based quality improvement program. Ann Otol Rhinol Laryngol.

[26] Wall LR, Kularatna S, Ward EC, Cartmill B, Hill AJ, Isenring E, Byrnes J, Porceddu SV (2019). Economic analysis of a three-arm RCT exploring the delivery of intensive, prophylactic swallowing therapy to patients with head and neck cancer during (chemo)radiotherapy. Dysphagia.

