# Supplementary material for: Evaluation of Mobile Health Technology Interventions for the Postdischarge Management of Patients With Head and Neck Cancer: Scoping Review
Source: JMIR Mhealth Uhealth. 2023 Oct 23;11:e49051. doi: 10.2196/49051 (PMC10628684; doi:10.2196/49051)
Supplement: Multimedia Appendix 1 [file mhealth_v11i1e49051_app1.docx]

**APPENDIX A: KEYWORD SEARCH STRATEGY**

| **Concepts** | **(P)** **Head and Neck tumor** | **(I) mHealth Intervention** |
| --- | --- | --- |
| Author Keywords | Head and Neck Tumor  Oromaxillofacial head and neck tumor  Oral Neoplasm  Cancer of The Oral Cavity  OC  HNC | mHealth  “m health”  eHealth  “e Health”  Telehealth  “mobile health”  Mobile app*  Mobile technology  Mobile phone  Smartphone*  Cellular phone*  Cell phone*  Tablet*  Personal digital assistant  PDA  Wearable  Biosensor*  Portable electronic application*  Portable software application*  Text messages  Phone app*  Cellphone app*  Telephone app*  Remote monitoring  Remote patient monitoring  Remote patient management  Remote biometric monitoring  Telenephro*  Tele-nephro*  Telecare  Telecommunication* |
| Subject Headings: MeSH | Head and Neck Neoplasms [MeSH]  Neoplasms, Head and Neck [MeSH]  Cancer of the Head and Neck [MeSH]  Head and Neck Cancer [MeSH]  Mouth Neoplasms [MeSH]  Neoplasm, Mouth [MeSH]  Oral Cancer [MeSH] | Telemedicine[MeSH]  Smartphone[MeSH]  Mobile applications[MeSH] |

**MEDLINE/PubMed Search Statement**

(head and neck tumor [tw] OR Oromaxillofacial head and neck tumor [tw] OR oral neoplasm [tw] OR cancer of the oral cavity [tw] OR HNC[tw] OR OC[tw] OR head and neck neoplasms [mesh] OR neoplasms, head and neck [mesh] OR cancer of the head and neck [mesh] OR head and neck cancer [mesh] OR mouth neoplasms [mesh] OR neoplasm, mouth [mesh] OR oral cancer [mesh]) AND (mHealth[tw] OR “m health”[tw] OR eHealth[tw] OR “e Health”[tw] OR telehealth[tw] OR “mobile health”[tw] OR mobile app*[tw] OR mobile technology[tw] OR mobile phone*[tw] OR smartphone*[tw] OR cellular phone*[tw] OR cell phone*[tw] OR tablet*[tw] OR personal digital assistant[tw] OR pda[tw] OR wearable[tw] OR biosensor*[tw] OR portable electronic application*[tw] OR portable software application*[tw] OR text messages[tw] OR phone app*[tw] OR cellphone app*[tw] OR telephone app*[tw] OR remote monitoring[tw] OR remote patient monitoring[tw] OR remote patient management[tw] OR remote biometric monitoring[tw] OR telenephro*[tw] OR tele-nephro*[tw] OR telecare[tw] OR telecommunication*[tw] OR telemedicine[MeSH] OR smartphone[MeSH] OR mobile applications[MeSH])

**Web of science Search Statement**

Title: TI=(app or smartphone or “cell phone” or “mobile phone” or “mHealth” or “m health” or “eHealth” or “e Health” or “telehealth” or “mobile health” or “mobile app” or “ mobile technology” or “cellular phone” or “personal digital assistant” or “pda” or “wearable” or “ biosensor” or “portable electronic application” or “portable software application” or “text messages” or “phone app” or “cellphone app” or “telephone app” or “remote monitoring” or “remote patient monitoring” or “remote patient management” or “remote biometric monitoring” or “telenephro” or “tele-nephro” or “telecare” or “telecommunication” or “telemedicine” or “smartphone” or “mobile applications”) AND TI=:( head and neck tumor or Oromaxillofacial head and neck tumor or “oral neoplasm” or “cancer of the oral cavity” or “HNC” or “OC” or “head and neck neoplasms” or “cancer of the head and neck” or “head and neck cancer” or “mouth neoplasms” or “oral cancer”) OR Abstract: AB=( app or smartphone or “cell phone” or “mobile phone” or “mHealth” or “m health” or “eHealth” or “e Health” or “telehealth” or “mobile health” or “mobile app” or “ mobile technology” or “cellular phone” or “personal digital assistant” or “pda” or “wearable” or “ biosensor” or “portable electronic application” or “portable software application” or “text messages” or “phone app” or “cellphone app” or “telephone app” or “remote monitoring” or “remote patient monitoring” or “remote patient management” or “remote biometric monitoring” or “telenephro” or “tele-nephro” or “telecare” or “telecommunication” or “telemedicine” or “smartphone” or “mobile applications”) AND AB=:( head and neck tumor or Oromaxillofacial head and neck tumor or “oral neoplasm” or “cancer of the oral cavity” or “HNC” or “OC” or “head and neck neoplasms” or “cancer of the head and neck” or “head and neck cancer” or “mouth neoplasms” or “oral cancer”)

**Embase Search Statement**

('head and neck tumor'/exp OR 'tongue cancer'/exp OR 'mouth cancer'/exp OR 'mouth tumor'/exp OR 'oromaxillofacial head and neck tumor' OR 'head and neck cancer'/exp) AND (mhealth OR 'telehealth'/exp OR 'mobile health technology'/exp OR 'mobile phone'/exp OR 'smartphone'/exp OR cellphone OR 'tablet'/exp OR 'pda'/exp OR 'personal digital assistant'/exp OR 'wearable device'/exp OR 'telemedicine'/exp OR 'mobile application'/exp OR 'telecare'/exp OR 'phone app' OR 'text messages' OR 'remote monitoring system'/exp)

**CINAHL Search Statement**

head and neck tumor OR Oromaxillofacial head and neck tumor OR“oral neoplasm ”OR “cancer of the oral cavity” OR "HNC" OR " OC " OR “head and neck neoplasms” OR “cancer of the head and neck” OR “head and neck cancer” OR “mouth neoplasms” OR “oral cancer” **AND** mHealth OR “m health” OR eHealth OR “e Health” OR telehealth OR “mobile health” OR “mobile app*” OR “mobile technology” OR “mobile phone*” OR smartphone* OR “cellular phone*” OR “cell phone*” OR tablet* OR “personal digital assistant” OR “PDA” OR wearable OR biosensor* OR “portable electronic application*” OR “portable software application*” OR “text messages” OR “phone app*” OR “cellphone app*” OR “telephone app*” OR “remote monitoring” OR “remote patient monitoring” OR “remote patient management” OR “remote biometric monitoring” OR telenephro* OR tele-nephro* OR telecare OR telecommunication* OR telemedicine
