# Supplementary material for: Evaluation of Mobile Health Technology Interventions for the Postdischarge Management of Patients With Head and Neck Cancer: Scoping Review
Source: JMIR Mhealth Uhealth. 2023 Oct 23;11:e49051. doi: 10.2196/49051 (PMC10628684; doi:10.2196/49051)
Supplement: Multimedia Appendix 2 [file mhealth_v11i1e49051_app2.docx]

| Source | Software name or type | Development purpose and function | Participants | Interventions | | Results and conclusions |
| --- | --- | --- | --- | --- | --- | --- |
| Ma, D et al, 2021 | Northwell Head & Neck Health Chats | Get access to health and medical information at any time and report pro adverse outcomes  (1) Symptoms reporting  (2) Chat feedback | HNC:233  intervention group: n=84  control group: n=138 | 4 months.  The intervention will be sent to patients 2 days before each weekly treatment visit.  During the first 4 weeks after treatment, the intervention will be sent weekly.  During the 4-month follow-up period, the intervention will be sent monthly. | | 1.The consistency between PRO and clinical doctor reported outcomes (CRO) is good, with a range of 0.10 to 0.43.  2.The most common symptoms are sialorrhea (53%), xerostomia (41%), and gingivitis (37%).  3.Eighty-nine percent (39 of 44) of respondents found the chat software easy to use, and 61% reported that it helped with self-management of symptoms and reduced the need for call-backs to the care team.  An interactive chatbot is feasible and can provide support to HNC patients during and after RT. |
| Wall, L. et al 2020 | SwallowIT | Comparison of the clinical efficacy of three models for providing preventive swallowing treatment during chemotherapy and radiotherapy.  (1) Provide educational videos, images, and other media materials.  (2) Identify and record patient training data. | HNC：79  26/26/27 | 6 weeks.  Each intervention is repeated 10 times, with 1 intervention per day (10 interventions per cycle = 5 repetitions per intervention).  Patients receive 6 interventions per week, with the first week being RT (radiation therapy). | | All groups had a low compliance (27%) at 6 weeks and the compliance started to decrease from the fourth week of (C)RT. The compliance of the clinical doctor-supervised group was significantly better than the patient self-management group. At the first to third weeks, the compliance of the SwallowIT group was also higher than that of the patient guidance group. There were no significant differences in swallowing, nutrition, or function measurements.  In the patient guidance group, 76% preferred SwallowIT and/or clinical doctor guidance.  In the case of similar clinical outcomes to traditional care models, patients preferred SwallowIT and clinical doctor guidance as treatment, possibly due to the higher level of treatment support available. |
| Graboyes, E. M.et al,2020 | BRIGHT | BRIGHT focuses on adapting to physical changes and functional changes, teaching skills for coping and problem-solving, and aiming to reduce avoidant behavior in patients with BID.  (1) Psychological education about appearance issues and internal beliefs about appearance.  (2) Introduction of cognitive models and cognitive restructuring.  (3) Avoidance behavior and social support.  (4)Identification of personal values. | Oral cancer:10 | | 3 months  Five 60-minute video conferences per week | The remote medical platform operates well and the overall satisfaction is high.  The body image scale average score decreased by 5.1% after 3 months of BRIGHT, and this effect persisted for 3 more months after BRIGHT.  BRIGHT is feasible and accepted by HNC survivors, and it can be a new method for managing BID in HNC survivors. |
| Van Cleave, J. H et al,2021 | ePVA | developed for patients to report their symptoms and functional limitations.  Questions about 21 common symptoms and functional status limitations are asked and recorded, and an ePVA report is generated. The HNC care team uses the ePVA report to intervene clinically in real time based on their clinical judgment and the knowledge of participants. | HNC：75 | | 6 months  In most cases, participants who receive 7-week combined chemotherapy and radiotherapy can complete the ePVA at 2 to 3 time points. | Participants who reported the highest number of symptoms and functional limitations also reported significantly lower overall EORTC QLQ-C30 QoL/health scores  Participants with current health limitations in social activities had the lowest average EORTC QLQ-C30 global QoL/health scores  ePVA may be a useful mHealth tool that as a clinical support tool for real-time interventions for patient-reported symptoms and HNC functional limitations. |
| Wang, T. F. et al,2020 | Mobile app | Support for patients with message alerts, symptom recording, access to medical health information and follow-up reminders  1. Latest news  2. Medical information  3. Self-record  4. Follow up reminders | Oral cancer：100  intervention group: n=50  control group: n=50 | | 3 months  No regular frequency | Physical care needs were significantly lower in the trial group compared to the control group (p<.05)  Psychological needs, communication needs and care support needs all improved after the mHealth app intervention Overall improvement in quality of life was higher in the experimental group than in the control group (-7.24 vs -4.36)  For post-operative patients, the educational/informational intervention using the mHealth app significantly  reduced their care needs, improved their quality of life and increased their acceptance of using the mHealth App on their mobile devices |
| Di R; Li, G,2018 | Mobile app | Interventions for patients discharged from hospital with nasopharyngeal carcinoma who have received radiotherapy, sharing information on nasopharyngeal carcinoma disease, the  such as observation and treatment of radiotherapy complications, and regular review of  To improve the self-management skills of discharged patients so that they can effectively cope with radiotherapy complications and improve their quality of life  1. Review reminders  2. Medical knowledge base  3. Online experts | nasopharyngeal carcinoma：132  intervention group: n=67  control group: n=65 | | 6 months  Patients are notified 3 days prior to their review  Every Wednesday and Saturday, the doctor is scheduled to answer patients' questions online | The differences in radiotherapy complications and quality of life at discharge were not statistically significant.  In the intervention group, 5 months after discharge, the incidence of oral mucositis, xerostomia, difficulty in opening the mouth and  The incidence of nasal congestion was significantly lower and the quality of life was significantly higher in the intervention group than in the control group.  Appropriate use of this smartphone app can improve nasopharyngeal cancer patients discharged from hospital  exercise compliance in receiving radiotherapy, reduce the adverse effects and complications of radiotherapy, and improve the quality of life and satisfaction of patients after discharge. |
| van den Brink, J. L. et al,2007 | Tablets | Providing medical health information and telemedicine support  1. Communication (sending messages)  2. Access to medical and health information  3. Contact with other patients (via forum)  4. Monitoring at home (via electronic questionnaires) | HNC：184  intervention group: n=39  control group: n=145 | | 6 weeks  Information support category, no intervention  In the monitoring module: ranging from twice a week - once every 4 weeks | At the end of 6 weeks of the intervention, the intervention group had significantly improved QoL in 22 of the 5 study parameters.  Only one of these five quality of life parameters remained significantly different at 12 weeks  Telemedicine may be beneficial for quality of life in cancer patients. |
| Lin, T. et al,2022 | Oral Care Mobile APP | Providing medical health information and advice on oral mucosal care  1. Educational forums (media materials)  2. Self-recording (symptoms)  3. Oral mucosa care advice  4. Discussion platform (visual images can be viewed, interactive) | HNC：84  intervention group: n=41  control group: n=43 | | Pre-treatment - 2 months post-treatment  No fixed intervention frequency | PG-SGA scores were significantly lower in the intervention group than in the control group at all three time points.  Hb and albumin decreased less in the intervention group than in the control group at 2 months. The severity of oral mucositis grading was significantly lower in the intervention group than in the control group at all three time points.  Use of a mobile phone app was effective in improving synchronous radiotherapy of head and neck cancer patients, reduce side effects and improve quality of life. |
| Pfeifer, M. P. et al,2015 | Health buddy | Patients respond according with self-reported symptoms or behaviours and visit the coordinator electronically on a daily basis through the study.  Symptom interventions that do not resolve or require immediate relief will result in the coordinator contacting the patient directly by telephone and/or contacting the clinician to ensure effective and immediate intervention.  Use of a landline telephone and a simple A four-button keypad to answer daily questions | HNC：80  intervention group: n=45  control group: n=35 | | 3 weeks  Answered daily, with a daily response time of 5-10 minutes. | In the post-treatment phase, telemedicine participants had significantly higher physical health scores than the control group  The use of telemedicine to support head and neck patients in the acute phase of cancer treatment improved some aspects of post-treatment quality of life and symptom burden. |
| van der Hout, A. et al,2020 | Oncokompas | monitoring generic and tumour-specific symptoms of cancer and HRQOL. provide feedback and information on scores and a personalised overview of supportive care options | HNC：185  intervention group: n=99  control group: n=86 | | 6 months  No fixed intervention frequency  Automatically generated reminders sent every 3 months to encourage repeat use of Oncokompas | There was a statistically significant improvement in the clinical course of oral pain, social eating, swallowing, coughing and dental symptoms in the intervention group compared to the control group.  Oncokompas did not improve knowledge, skills and confidence in self-management or other secondary outcome indicators (e.g. supportive care needs). Only secondary outcomes of HRQOL and tumour-specific symptom burden improved. |
| Starmer, H. M. et al,2022 | HNC Virtual Coach | Preventive swallowing rehabilitation mobile app | HNC: 91  intervention group: n=44  control group: n=47 | | 3 months  Three sets of ten repetitions of four different exercises, twice a day:  Jaw extension, effortful swallowing, Mendelssohn action movements | Patients using the HNC Virtual Coach tended to comply better with treatment recommendations during radiation therapy.  Increased compliance was associated with better patient-reported quality of life, but not with physiological functioning 2-3 months after completion of radiotherapy.  The mHealth app may provide benefits for some patients receiving head and neck radiation therapy. |
| Shah, M. et al,2020 | telephone | Post-operative telephone survey and use of wound care telephone  Post-operative telephone follow-up to initiate video or wound pictures to reduce readmission rates | HNC:169  intervention group: n=78  control group: n=91 | | 72 hours after surgery  A survey of the situation based on a questionnaire and the possibility of sending pictures or videos of the wound for visits and examinations and answering questions from patients | Two patients were readmitted, two of whom could not be contacted. Patients who could not be contacted were readmitted for dysphagia and urinary tract infection . Patients who were contacted were advised to go to the emergency department during calls concerned about post-operative bleeding and gastrointestinal bleeding. 7 patients used the wound care telephone. The telephone survey prevented them from going to the emergency department. There was a statistically significant reduction in emergency department visits compared to the previous year and no change in readmission rates.Implementing telephone calls early in the post-operative period has the potential to reduce unnecessary emergency room visits and increase patient satisfaction. This can reduce pressure on the healthcare system and improve patient care. |
| Wang, T. J. et al,2019 | telephone | Provide additional support to improve adherence  Monitor training progress  addressing questions and concerns.  Provide feedback on training  Motivating adherence | Oral cancer:60  intervention group: n=30  control group: n=30 | | 3 months  The base intervention was the same, with the interventionist calling each subject at weeks 1, 2, 3, 4, 8 and 12 to monitor training progress, improve adherence and address any questions and concerns. | The findings support the effectiveness of remote support via telephone to enhance compliance with the intervention programme and the effectiveness of the intervention programme in reducing dental and jaw function impairment in patients undergoing radical oral cancer surgery. |
