# Supplementary material for: Evaluation of Mobile Health Technology Interventions for the Postdischarge Management of Patients With Head and Neck Cancer: Scoping Review
Source: JMIR Mhealth Uhealth. 2023 Oct 23;11:e49051. doi: 10.2196/49051 (PMC10628684; doi:10.2196/49051)
Supplement: Multimedia Appendix 3 [file mhealth_v11i1e49051_app3.docx]

**Multimedia Appendix3. JBI’s Critical appraisal tools**

**Table 1.** The methodological quality of the included randomized controlled trials.

| Study | Scoring (yes, ?, or no) | 1: Randomization | 2: Treatment allocation | 3: Group similarity | 4: Blinding of participants | 5: Blinding of treatment delivery | 6: Blinding of outcome assessors | 7: Treatment groups treated identically | 8: Follow-up | 9: Participants analyzed in groups | 10: Outcome assessment similarity | 11: Outcome assessment reliability | 12: Statistical analysis | 13: Trial design or other deviations |
| --- | --- | --- | --- | --- | --- | --- | --- | --- | --- | --- | --- | --- | --- | --- |
| van der Hout, A.et al,2020 | 9/1/3 | ^a^Y | Y | Y | ^b^N | N | N | Y | ^c^? | Y | Y | Y | Y | Y |
| Starmer, H. M. et al,2022 | 10/3/0 | Y | Y | Y | ^b^? | ? | ? | Y | ? | Y | Y | Y | Y | Y |
| Wang, T. J. et al,2019 | 13/0/0 | Y | Y | Y | Y | Y | Y | Y | Y | Y | Y | Y | Y | Y |
| Pfeifer, M. P. et al,2015 | 9/4/0 | Y | Y | Y | ? | ? | ? | Y | Y | ? | Y | Y | Y | Y |
| Di R; Li, G,2018 | 10/3/0 | Y | Y | Y | ? | ? | ? | Y | Y | Y | Y | Y | Y | Y |
| Wall, L. et al 2020 | 11/0/2 | Y | Y | Y | N | N | Y | Y | Y | Y | Y | Y | Y | Y |

^a^Y: yes.

^b^N: no.

^c^?: unclear.

**Table 2.** The methodological quality of the included cohort studies.

| Study, year | Scoring (yes, ?, or no) | 1: Group similarity and recruitment | 2: Exposure similarity | 3: Exposure validity and reliability | 4: Confounding factors | 5: Strategies for confounding factors | 6: Baseline outcome situation | 7: Outcome assessment reliability | 8: Follow-up time | 9: Follow-up completion | 10: Strategies for completion | 11: Statistical analysis |
| --- | --- | --- | --- | --- | --- | --- | --- | --- | --- | --- | --- | --- |
| Van Cleave, J. H et al,2021 | 2/6/3/0 | N/A^a^ | N/A | Y^b^ | ?^c^ | ? | Y | Y | Y | Y | ? | Y |
| Ma, D et al, 2021 | 3/5/1/2 | N/A | N/A | N/A | N^d^ | N | Y | Y | Y | Y | ? | Y |

^a^N/A: not applicable.

^b^Y: yes.

^c^?: unclear.

^d^N: no.

**Table 3.** The methodological quality of the included non-randomized controlled trial.

| Study, year | Scoring (yes, ?,or no) | 1: Clear cause and effect | 2: Comparison of similarity | 3: Participants included in any comparisons receiving similar treatment | 4: Control group | 5: Pre- and postintervention assessments | 6: Follow-up | 7: Outcome assessment similarity | 8: Outcome assessment reliability | 9: Statistical analysis |
| --- | --- | --- | --- | --- | --- | --- | --- | --- | --- | --- |
| Lin, T. et al,2022 | 8/1/0/0 | Y^a^ | Y | Y | Y | Y | ?^b^ | Y | Y | Y |
| van den Brink, J. L. et al,2007 | 9/0/0/0 | Y | Y | Y | Y | Y | Y | Y | Y | Y |
| Wang, T. F. et al,2020 | 9/0/0/0 | Y | Y | Y | Y | Y | Y | Y | Y | Y |
| Graboyes, E. M.et al,2020 | 6/0/3/0 | Y | N | N | N | Y | Y | Y | Y | Y |
| Shah, M. et al,2020 | 4/0/2/3 | Y | N/A^d^ | N/A | N | N | Y | N/A | Y | Y |

^a^Y: yes.

^b^?: unclear.

^c^N: no.

^d^N/A: not applicable.
